# 肿瘤内微生物群与肺癌关系的研究进展

**DOI:** 10.3779/j.issn.1009-3419.2025.101.05

**Published:** 2025-04-20

**Authors:** Yangtong ZHU, Jiawei CHEN, Yanqian ZHU, Linyu WU

**Affiliations:** 310006 杭州，浙江中医药大学附属第一医院（浙江省中医院），浙江中医药大学第一临床医学院; The First Affiliated Hospital of Zhejiang Chinese Medical University (Zhejiang Provincial Hospital of Chinese Medicine), First Clinical School of Medicine, Zhejiang Chinese Medical University, Hangzhou 310006, China

**Keywords:** 肺肿瘤, 瘤内微生物, 作用机制, 生物标志物, Lung neoplasms, Intratumor microbiota, Mechanism of action, Biomarker

## Abstract

肺癌的发病率和死亡率在全球癌症中居于首位。近年来，随着微生物检测技术的发展，肿瘤内微生物群逐渐成为肺癌研究领域的热点和前沿。研究发现存在于肿瘤内的微生物群可以通过多种方式影响肺癌的发生发展。此外，肿瘤内微生物群可以作为肺癌诊断和预后评估的潜在生物标志物，通过调节肺癌瘤内微生物群有望成为新型的肺癌治疗方式。本文将系统综述肿瘤内微生物群与肺癌相关的最新研究进展，总结肺癌瘤内微生物群的来源和特征，探讨其影响肺癌发生发展的机制，并展望其在肺癌诊断、治疗和预后中的应用前景。

## Abstract (en)

Lung cancer has the highest incidence and mortality of any cancer in the world. In recent years, with the development of microbial detection technology, the intratumor microbiota has gradually become a hot spot and frontier in the field of lung cancer research. Studies have found that the microbiota present in tumors can influence the development of lung cancer in a variety of ways. In addition, the intratumor microbiota can be used as a potential biomarker for the diagnosis and prognosis assessment of lung cancer, and the regulation of the intratumor microbiota of lung cancer is expected to become a new type of lung cancer treatment. In this paper, we reviewed the latest research progress on the relationship between intratumor microbiota and lung cancer, summarized the origin and characteristics of intratumor microbiota, discussed the mechanism of its influence on the occurrence and development of lung cancer, and explored its potential applications in the diagnosis, treatment and prognosis of lung cancer.

根据国际癌症研究机构（International Agency for Research on Cancer, IARC）发布的2022年度全球癌症统计数据^[1]^显示，肺癌是全球范围内发病率和死亡率最高的恶性肿瘤，在全球有近250万例新增病例和超过180万例死亡病例，分别占全球癌症诊断的12.4%和癌症死亡的18.7%。并且在大多数国家，肺癌患者的5年生存率仅为10%-20%^[2]^。因此，提高肺癌患者的预后水平至关重要。微生物在当代肺癌研究中扮演着关键角色，能够影响癌症的多种生物学行为^[3]^。随着16S rRNA基因测序等高通量技术的应用，越来越多的研究^[4-7]^发现肺癌内存在共生微生物群。目前的研究^[3]^表明，肿瘤内微生物群可通过介导慢性炎症、调控免疫反应、调节代谢变化等多种途径参与肺癌的发生发展，也可作为肺癌诊断和预后评估的新兴生物标志物以促进患者的个性化诊疗。基于肿瘤内微生物群与肺癌的密切关系，现就其在肺癌发生发展、肺癌诊断、治疗以及预后评估中的研究进展进行阐述。

## 1 肺癌瘤内微生物群的概述

肿瘤内微生物群是指存在于肿瘤组织内并构成肿瘤微环境（tumor microenvironment, TME）的微生物群，包括细菌、病毒、真菌和其他真核生物^[8,9]^。100多年前研究者^[9]^首次在人类肿瘤内发现了细菌，但当时的技术手段和认知水平限制了人们进行深入研究。随着生物技术发展至今，多项证据表明多种微生物存在于肺癌组织内且与肺癌息息相关。Apostolou等^[10]^使用逆转录-聚合酶链反应在肺癌患者的组织样本中检测出了表皮葡萄球菌（Staphylococcus epidermidis）等细菌。Liu等^[5]^在肺腺癌（lung adenocarcinoma, LUAD）患者中发现了聚多曲霉（Aspergillus sydowii）的富集及其促进肺癌发展的机制。此外，研究^[11]^证实相比于免疫细胞、基质细胞等，存在于肿瘤细胞内的瘤内细菌负荷最高。

随着微生物群成为肺癌研究领域的热点，研究者们对肺癌瘤内微生物的来源进行了广泛探讨。目前的研究认为可能有3个主要来源^[3,8,12]^。第一，通过肺黏膜屏障浸润肿瘤。在肿瘤发生的过程中，存在于肺黏膜上的微生物可能在黏膜屏障破坏后浸润肿瘤^[8]^，但仍需要更多的研究来确认微生物从黏膜进入TME的机制^[12]^。第二，来源于正常邻近组织。由于肿瘤组织与正常邻近组织细菌组成的高度相似性，肿瘤内细菌可能源于正常邻近组织^[4]^。然而，另有研究^[12]^提出，正常邻近组织中的微生物可能来自于TME。第三，其他部位的微生物通过血液循环传播到肺部肿瘤组织。Zhu等^[13]^通过对Lewis肺癌小鼠模型的粪便、血液和肿瘤组织样本分析发现，与对照组小鼠相比，嗜黏蛋白阿克曼氏菌（Akkermansia muciniphila, Akk）灌胃的小鼠血液循环和肿瘤组织中的Akk丰度增加，提示肠道微生物可能迁移到血液循环中，随后定植到肺癌组织中。

近年来，研究^[14-16]^表明肺癌组织中的微生物群落特征与正常肺组织存在显著差异，在肺癌组织中，微生物的丰度和多样性通常低于对应的正常肺组织。而在不同亚型的肺癌之间，其肿瘤组织内的菌群组成也具有特异性差异。例如，TP53突变阳性患者的肺鳞癌（lung squamous cell carcinoma, LUSC）组织中食酸菌属（Acidovorax）表现出了更高的丰度，而在LUAD中未出现这种关联^[6]^。与LUSC相比，LUAD组织中罗尔斯通菌属（Ralstonia）相对丰度更低，而栖热菌属（Thermus）相对丰度更高^[7]^。该研究^[7]^还指出，栖热菌属在IIIB、IV期等晚期肺癌患者的肺癌组织中显著富集，而军团菌在发生转移的肺癌组织中丰度更高，表明瘤内微生物群在不同肺癌分期中的优势菌群也不同。总之，这些发现不仅为深入理解肺癌的发病机制提供了新的视角，也为未来肺癌个性化诊疗提供了新的思路。

## 2 肿瘤内微生物群影响肺癌发生发展的潜在作用机制

随着肿瘤内微生物群与肺癌的相关性逐渐成为研究热点，其作用机制正不断被探索。肿瘤内微生物群主要通过炎症介导、免疫调节、代谢调节等机制影响肺癌。

### 2.1 介导慢性炎症

肿瘤内微生物群能够通过调节炎症因子的产生在肺部形成慢性炎症微环境从而促进肺癌的发生发展。在肺癌中，局部细菌负荷的增加和菌群组成的改变可以刺激骨髓细胞产生Myd88依赖性白细胞介素-1β（interleukin-1β, IL-1β）和IL-23等细胞因子的表达，进一步促进Vγ6^+^Vδ1^+^γδ T细胞的增殖和激活，使其产生释放IL-17和IL-22，从而促进炎症和肿瘤生长^[17,18]^。既往研究已发现温和食酸菌（Acidovorax temperans, A. temperans）富集于肺癌组织中，Stone等^[19]^将A. temperans多次鼻内滴注到K-ras和TP53突变阳性的LUAD小鼠中，发现A. temperans暴露在肺癌小鼠模型中引起成熟的肿瘤相关中性粒细胞分泌集落刺激因子1，以促进单核细胞向巨噬细胞分化，进而上调主要组织相容性复合体（major histocompatibility complex, MHC）II类分子表达，以刺激T细胞进入T17极化，引起肿瘤相关炎症的产生，为菌群失调如何影响肺癌发展提供了机制见解。此外，Elkrief等^[20]^进行的一项探索性转录组学分析揭示了埃希氏菌（Escherichia）与非小细胞肺癌（non-small cell lung cancer, NSCLC）样本中T细胞激活相关途径的富集有关，提示肿瘤内埃希氏菌可能同样参与促进炎症性TME的形成。这些研究共同揭示了肺癌瘤内微生物群的负荷和组成的改变对肺癌产生重要影响，可以通过调节炎症因子导致的慢性炎症微环境进而促进肿瘤细胞的增殖。

### 2.2 调节免疫反应

瘤内微生物能够发挥免疫调节作用促进肺癌生长，主要是通过激活免疫抑制细胞，如肿瘤相关巨噬细胞、髓源性抑制细胞、调节性T细胞和肿瘤相关中性粒细胞，同时抑制T细胞和自然杀伤（natural killer, NK）细胞等抗肿瘤免疫细胞的激活和增殖^[21]^。Liu等^[5]^研究发现肿瘤组织中的聚多曲霉及其衍生的β-葡聚糖可以促进分泌IL-1β并诱导髓源性抑制细胞聚集，抑制细胞毒性T淋巴细胞的活性并促进调节性T细胞的聚集，形成免疫抑制微环境；Shen等^[22]^使用16S rRNA基因测序对肺癌组织进行微生物分析，发现蓝细菌（Cyanobacteria）等特定微生物群的相对丰度与CD8^+^ T细胞的浸润程度和程序性细胞死亡受体1的表达水平呈正相关，表明肺癌内的微生物可能参与塑造肿瘤免疫抑制微环境从而促进肺癌生长。值得注意的是，共生菌也在维持宿主免疫稳态和抗肿瘤免疫反应中有着积极作用。Cheng等^[23]^研究发现经过抗生素处理的小鼠平均生存时间减少且肺部肿瘤数量增加、体积增大，且更容易发生B16/F10黑色素瘤和Lewis肺癌转移，但在添加正常γδT细胞或补充IL-17之后可恢复小鼠受损的免疫应答，表明共生菌可通过γδT17免疫细胞依赖性机制调节宿主对肺肿瘤细胞的免疫监视功能。以上研究表明瘤内微生物在肺癌生长中的免疫调节作用具有双重性，既可能通过形成免疫抑制微环境促进肿瘤进展，也可能通过调节免疫稳态来抑制肿瘤发展。

### 2.3 调节代谢途径

肺癌瘤内微生物除了参与调节炎症和免疫反应之外，也可通过调节宿主代谢途径发挥作用。Apopa等^[24]^将LUAD组织中蓝藻及其代谢物微囊藻毒素的存在与CD36减少和多聚ADP核糖聚合酶1[poly(ADP-ribose) polymerase 1, PARP1]增加联系起来，结果表明微囊藻毒素可以激活肺癌发生相关的炎症途径进而促进肺癌发展。而通过控制肺癌组织中蓝藻样颗粒或微囊藻毒素的流入以及抑制PARP1可以防治炎症相关的肺癌。有研究^[25]^向皮下移植肺癌的小鼠瘤内注射产丁盐酸的肠道罗斯拜瑞氏菌（Roseburia intestinalis），相比于空白对照组，罗斯拜瑞氏菌组小鼠不仅肿瘤体积显著增加且发生了肺癌转移，而且较低浓度的丁酸盐可能增强肿瘤细胞的迁移和侵袭能力，使它们更容易实现远处转移。这项研究揭示了肠道罗斯拜瑞氏菌可通过产生丁酸盐来上调H19启动子表达并促进M2巨噬细胞极化从而促进肺癌转移，提示瘤内微生物群也可参与调节肺癌转移。另有研究^[26]^发现肺炎链球菌（Streptococcus pneumoniae）通过将肺炎球菌表面蛋白C（pneumococcal surface protein C, PspC）与血小板活化因子受体（platelet-activating factor receptor, PAFR）结合而附着在肺癌细胞上。PspC和PAFR之间的相互作用刺激细胞增殖并激活PI3K/AKT和核因子κB（nuclear factor kappa-B, NF-κB）信号通路，在肺部发挥致癌作用。总之，肺癌瘤内微生物通过复杂的代谢调节机制，包括影响炎症途径、巨噬细胞极化和信号通路激活等，在肺癌的发生和发展中发挥着关键作用。

## 3 肺癌瘤内微生物群的临床应用前景

### 3.1 肺癌诊断

已有研究发现肺癌组织和正常肺组织之间以及不同肺癌组织之间的微生物群具有显著差异，这可能有助于肺癌诊断。研究^[27]^发现，与非肿瘤组织相比，肺癌组织中发现普雷沃氏菌属（Prevotella）、双歧杆菌属（Bifidobacterium）等丰度更高，而食酸菌属、不动杆菌属（Acinetobacter）等丰度较低。与良性肿块样病变患者相比，肺癌患者韦荣氏球菌属（Veillonella）和巨球型菌属（Megasphaera）的丰度较高，显示出了作为诊断性生物标志物的潜力^[28]^。一项真菌组分析^[29]^在肺癌组织中发现了芽生菌（Blastomyces），可以为真菌作为生物标志物以区分肺癌和健康患者提供依据。Uzelac等^[30]^评估了LUAD、LUSC和健康个体肺组织样本中的古细菌物种丰度，开发了一种基于机器学习的预测算法，能够以99%的准确率区分肺癌患者和健康个体。然而，该方法需要已知癌症发病率并且须通过提取肿瘤组织进行分析比较，因此这种诊断方式仍有限制。目前肿瘤内微生物群与肺癌诊断相关方面的研究相对较少，今后期待有更多的研究来明确瘤内微生物群作为生物标志物在肺癌诊断中的地位并促进其临床应用。

### 3.2 肺癌治疗

目前常见的肺癌治疗方案包括手术、化疗、放疗和免疫治疗等，而瘤内微生物能通过多种机制影响肺癌进展，有望成为肺癌治疗的潜在靶点。在癌症治疗中，可以通过两种方法来调整肿瘤内微生物群，包括消除无益但丰富的微生物和补充有益但减少的微生物^[3]^。但在有关肺癌治疗方面的研究中，这两种方法的应用仍然较少。鉴于抗生素可能引起菌群失衡以及细菌耐药性的增加，研究者正在探索将噬菌体作为靶向调节肿瘤内细菌的生物制剂^[31]^。靶向噬菌体及其对应的靶向肽显示出了仅针对肺癌细胞系及临床样本的高度亲和力，而未对正常肺组织表现出类似效应，从而提高药物递送的精确性，提升肺癌治疗效果^[32]^。肿瘤内微生物在肺癌治疗方面的研究虽有积极结果，但相关研究较少且尚不能转化为临床疗法。因此，深入研究其作用机制有望为肺癌治疗提供新的治疗思路和策略。

### 3.3 肺癌预后评估

肺癌瘤内微生物群可能与肺癌患者的不良预后存在潜在关联，被视为一种有前景的肺癌预后生物标志物。研究者发现聚多曲霉的富集与患者的侵袭性病理和淋巴结浸润有关，并且聚多曲霉的丰度可在总生存期和无进展生存期方面对肺癌患者进行分层^[5]^。Peters等^[33]^进行的微生物群分析结果显示，肿瘤组织中较高丰度的放线菌目（Actinomycetales）和假单胞菌目（Pseudomonadales）与较差的无病生存期相关，正常肺组织中较高丰度的拟杆菌目（Bacteroidales）和梭菌目（Clostridiales）与较差的无复发生存期相关。在接受一线治疗的NSCLC样本中，副流感嗜血杆菌（Haemophilus parainfluenzae）、粘质沙雷氏菌（Serratia marcescens）、荣氏不动杆菌（Acinetobacter jungii）、星座链球菌（Streptococcus constellation）这4种细菌的Logistic回归分析能够有效地预测患者2年生存率，其准确率可达到90.7%^[34]^。而消化球菌属（Peptococcus）则被确认为是一个独立且负性的NSCLC预后因素，可通过肿瘤坏死因子信号传导引起不良的预后结果^[35]^。这些初步结果表明，来自肺部微生物群的新型生物标志物有望预测肺癌患者的预后。此外，Zhou等^[36]^发现LUSC中复发或转移组和非复发转移组瘤内微生物群的丰富度具有显著差异，且受试者的宿主基因表达与肿瘤组织微生物组成之间存在显著联系（P=0.004）。基于此，他们建立了一个新型多模态机器学习模型以预测LUSC患者的复发或转移，其受试者工作特征曲线下面积可达0.81。这些研究结果表明，肿瘤组织中的微生物群有望成为预测肺癌复发或转移风险的有效工具，这或将有助于改善肺癌患者的生存结局。

## 4 小结与展望

随着高通量测序技术的持续发展，肺癌瘤内微生物群的存在已被证实，且有研究发现瘤内微生物群可以通过介导慢性炎症、调节免疫反应、调节代谢途径等多种途径参与肺癌的发生发展。但目前应用微生物进行肺癌诊断及治疗的临床转化仍然面临诸多的挑战。此外，由于肿瘤内微生物群生物量相对较低，导致肿瘤内微生物的检测也较困难，并且16S rRNA基因测序和鸟枪法宏基因组学可能会导致细菌DNA污染的问题也不容忽视^[37]^。因此，在分析肿瘤内微生物组时需要采取多种措施来避免或减少任何可能的污染，并且从多组学整合的角度探索肿瘤内微生物群与肺癌发生发展的相互作用，这将有助于为肿瘤内微生物的研究提供新的视角^[38]^。总之，肿瘤内微生物群的研究作为新兴热点仍处于起步阶段，未来需要研究者们更深入地探讨肿瘤内微生物群在肺癌发生发展中的具体机制，进一步促进其临床应用，从而为肺癌患者诊断、治疗及预后水平的提高提供可能性。

## References

[1] Bray F, Laversanne M, Sung H, et al. Global cancer statistics 2022: GLOBOCAN estimates of incidence and mortality worldwide for 36 cancers in 185 countries. CA Cancer J Clin, 2024, 74(3): 229-263. doi: 10.3322/caac.21834 38572751

[2] Allemani C, Matsuda T, Di Carlo V, et al. Global surveillance of trends in cancer survival 2000-14 (CONCORD-3): analysis of individual records for 37 513 025 patients diagnosed with one of 18 cancers from 322 population-based registries in 71 countries. Lancet, 2018, 391(10125): 1023-1075. doi: 10.1016/S0140-6736(17)33326-3 29395269 PMC5879496

[3] Liu W, Xu J, Pi Z, et al. Untangling the web of intratumor microbiota in lung cancer. Biochim Biophys Acta Rev Cancer, 2023, 1878( 6): 189025. doi: 10.1016/j.bbcan.2023.189025 37980944

[4] Nejman D, Livyatan I, Fuks G, et al. The human tumor microbiome is composed of tumor type-specific intracellular bacteria. Science, 2020, 368(6494): 973-980. doi: 10.1126/science.aay9189 32467386 PMC7757858

[5] Liu NN, Yi CX, Wei LQ, et al. The intratumor mycobiome promotes lung cancer progression via myeloid-derived suppressor cells. Cancer Cell, 2024, 42(2): 318-322. doi: 10.1016/j.ccell.2024.01.005 38350423

[6] Greathouse KL, White JR, Vargas AJ, et al. Interaction between the microbiome and TP 53 in human lung cancer. Genome Biol, 2018, 19(1): 123. doi: 10.1186/s13059-020-01961-0 PMC6109311 30143034

[7] Yu G, Gail MH, Consonni D, et al. Characterizing human lung tissue microbiota and its relationship to epidemiological and clinical features. Genome Biol, 2016, 17(1): 163. doi: 10.1186/s13059-016-1021-1 PMC4964003 27468850

[8] Yang L, Li A, Wang Y, et al. Intratumoral microbiota: roles in cancer initiation, development and therapeutic efficacy. Signal Transduct Target Ther, 2023, 8(1): 35. doi: 10.1038/s41392-022-01304-4 PMC9842669 36646684

[9] Gong Y, Huang X, Wang M, et al. Intratumor microbiota: a novel tumor component. J Cancer Res Clin Oncol, 2023, 149(9): 6675-6691. doi: 10.1007/s00432-023-04576-7 36639531 PMC11798032

[10] Apostolou P, Tsantsaridou A, Papasotiriou I, et al. Bacterial and fungal microflora in surgically removed lung cancer samples. J Cardiothorac Surg, 2011, 6: 137. doi: 10.1186/1749-8090-6-137 21999143 PMC3212932

[11] Wong-Rolle A, Dong Q, Zhu Y, et al. Spatial meta-transcriptomics reveal associations of intratumor bacteria burden with lung cancer cells showing a distinct oncogenic signature. J Immunother Cancer, 2022, 10(7): e004698. doi: 10.1136/jitc-2022-004698 PMC9260850 35793869

[12] Xie Y, Xie F, Zhou X, et al. Microbiota in tumors: from understanding to application. Adv Sci (Weinh), 2022, 9(21): e2200470. doi: 10.1002/advs.202200470 PMC9313476 35603968

[13] Zhu Z, Cai J, Hou W, et al. Microbiome and spatially resolved metabolomics analysis reveal the anticancer role of gut Akkermansia muciniphila by crosstalk with intratumoral microbiota and reprogramming tumoral metabolism in mice. Gut Microbes, 2023, 15(1): 2166700. doi: 10.1080/19490976.2023.2166700 PMC9904296 36740846

[14] Peters BA, Hayes RB, Goparaju C, et al. The microbiome in lung cancer tissue and recurrence-free survival. Cancer Epidemiol Biomarkers Prev, 2019, 28(4): 731-740. doi: 10.1158/1055-9965.EPI-18-0966 30733306 PMC6449216

[15] Mao Q, Ma W, Wang Z, et al. Differential flora in the microenvironment of lung tumor and paired adjacent normal tissues. Carcinogenesis, 2020, 41(8): 1094-1103. doi: 10.1093/carcin/bgaa044 32658980

[16] Liu HX, Tao LL, Zhang J, et al. Difference of lower airway microbiome in bilateral protected specimen brush between lung cancer patients with unilateral lobar masses and control subjects. Int J Cancer, 2018, 142(4): 769-778. doi: 10.1002/ijc.31098 29023689

[17] Jin C, Lagoudas GK, Zhao C, et al. Commensal microbiota promote lung cancer development via γδ T cells. Cell, 2019, 176(5): 998-1013.e16. doi: 10.1016/j.cell.2018.12.040 30712876 PMC6691977

[18] Liu Y, Han Y, Zeng S, et al. In respond to commensal bacteria: γδT cells play a pleiotropic role in tumor immunity. Cell Biosci, 2021, 11(1): 48. doi: 10.1186/s13578-021-00565-w PMC7927236 33653419

[19] Stone JK, von Muhlinen N, Zhang C, et al. Acidovorax temperans skews neutrophil maturation and polarizes Th 17 cells to promote lung adenocarcinoma development. Oncogenesis, 2024, 13(1): 13. doi: 10.1038/s41389-024-00513-6 PMC10991269 38570533

[20] Elkrief A, Montesion M, Sivakumar S, et al. Intratumoral escherichia is associated with improved survival to single-agent immune checkpoint inhibition in patients with advanced non-small-cell lung cancer. J Clin Oncol, 2024, 42(28): 3339-3349. doi: 10.1200/JCO.23.01488 39038258 PMC11600405

[21] Zhou Q, Zhou L, Chen X, et al. Crosstalk between the intratumoral microbiota and the tumor microenvironment: new frontiers in solid tumor progression and treatment. Cancer Rep (Hoboken), 2024, 7(11): e70063. doi: 10.1002/cnr2.70063 PMC11574561 39559964

[22] Shen S, Yu S, Yao D, et al. Special tissue microbiota such as Cyanobacteria are associated with the immune microenvironment of lung adenocarcinoma. Transl Cancer Res, 2024, 13(8): 4408-4419. doi: 10.21037/tcr-24-107 39262464 PMC11384924

[23] Cheng M, Qian L, Shen G, et al. Microbiota modulate tumoral immune surveillance in lung through a γδT 17 immune cell-dependent mechanism. Cancer Res, 2014, 74(15): 4030-4041. doi: 10.1158/0008-5472.CAN-13-2462 24947042

[24] Apopa PL, Alley L, Penney RB, et al. PARP 1 is up-regulated in non-small cell lung cancer tissues in the presence of the cyanobacterial toxin microcystin. Front Microbiol, 2018, 9: 1757. doi: 10.3389/fmicb.2018.01757 30127774 PMC6087756

[25] Ma Y, Chen H, Li H, et al. Intratumor microbiome-derived butyrate promotes lung cancer metastasis. Cell Rep Med, 2024, 5(4): 101488. doi: 10.1016/j.xcrm.2024.101488 PMC11031379 38565146

[26] Li N, Zhou H, Holden VK, et al. Streptococcus pneumoniae promotes lung cancer development and progression. iScience, 2023, 26(2): 105923. doi: 10.1016/j.isci.2022.105923 PMC9852931 36685035

[27] Liu Y, O'Brien JL, Ajami NJ, et al. Lung tissue microbial profile in lung cancer is distinct from emphysema. Am J Cancer Res, 2018, 8(9): 1775-1787. 30323970 PMC6176189

[28] Lee SH, Sung JY, Yong D, et al. Characterization of microbiome in bronchoalveolar lavage fluid of patients with lung cancer comparing with benign mass like lesions. Lung Cancer, 2016, 102: 89-95. doi: 10.1016/j.lungcan.2016.10.016 27987594

[29] Dohlman AB, Klug J, Mesko M, et al. A pan-cancer mycobiome analysis reveals fungal involvement in gastrointestinal and lung tumors. Cell, 2022, 185(20): 3807-3822.e12. doi: 10.1016/j.cell.2022.09.015 36179671 PMC9564002

[30] Uzelac M, Li Y, Chakladar J, et al. Archaea microbiome dysregulated genes and pathways as molecular targets for lung adenocarcinoma and squamous cell carcinoma. Int J Mol Sci, 2022, 23(19): 11566. doi: 10.3390/ijms231911566 PMC9570029 36232866

[31] Kabwe M, Dashper S, Bachrach G, et al. Bacteriophage manipulation of the microbiome associated with tumour microenvironments-can this improve cancer therapeutic response?. FEMS Microbiol Rev, 2021, 45(5): fuab017. doi: 10.1093/femsre/fuab017 33765142

[32] Chi YH, Hsiao JK, Lin MH, et al. Lung cancer-targeting peptides with multi-subtype indication for combinational drug delivery and molecular imaging. Theranostics, 2017, 7(6): 1612-1632. doi: 10.7150/thno.17573 28529640 PMC5436516

[33] Peters BA, Pass HI, Burk RD, et al. The lung microbiome, peripheral gene expression, and recurrence-free survival after resection of stage II non-small cell lung cancer. Genome Med, 2022, 14(1): 121. doi: 10.1186/s13073-022-01126-7 PMC9609265 36303210

[34] Zhang M, Zhang Y, Sun Y, et al. Intratumoral microbiota impacts the first-line treatment efficacy and survival in non-small cell lung cancer patients free of lung infection. J Healthc Eng, 2022, 2022: 5466853. doi: 10.1155/2022/5466853 35178229 PMC8844104

[35] Mao F, Hu Z, Shi R, et al. Unravelling the prognostic and operative role of intratumoural microbiota in non-small cell lung cancer: Insights from 16S rRNA and RNA sequencing. Clin Transl Med, 2025, 15(1): e70156. doi: 10.1002/ctm2.70156 PMC11702424 39754314

[36] Zhou X, Ji L, Ma Y, et al. Intratumoral microbiota-host interactions shape the variability of lung adenocarcinoma and lung squamous cell carcinoma in recurrence and metastasis. Microbiol Spectr, 2023, 11(3): e0373822. doi: 10.1128/spectrum.03738-22 PMC10269859 37074188

[37] Salter SJ, Cox MJ, Turek EM, et al. Reagent and laboratory contamination can critically impact sequence-based microbiome analyses. BMC Biol, 2014, 12: 87. doi: 10.1186/s12915-014-0087-z 25387460 PMC4228153

[38] Dai JH, Tan XR, Qiao H, et al. Emerging clinical relevance of microbiome in cancer: promising biomarkers and therapeutic targets. Protein Cell, 2024, 15(4): 239-260. doi: 10.1093/procel/pwad052 37946397 PMC10984626

